# The relationship between preoperative American Society of Anesthesiologists Physical Status Classification scores and functional recovery following hip-fracture surgery

**DOI:** 10.1186/s12891-017-1768-x

**Published:** 2017-10-10

**Authors:** Li-Huan Chen, Jersey Liang, Min-Chi Chen, Chi-Chuan Wu, Huey-Shinn Cheng, Hsiu-Ho Wang, Yea-Ing Lotus Shyu

**Affiliations:** 1 Department of Nursing, Yuanpei University of Medical Technology, 306 Yuanpei Street, Hsinchu, 30015 Taiwan; 2 Graduate Institute of Clinical Medical Sciences, Chang Gung University, 259 Wenhua 1st Road, Guishan District, Taoyuan, 33302 Taiwan; 3 Department of Health Management and Policy, School of Public Health, University of Michigan, 1415 Washington Heights, M3007 SPH II, Ann Arbor, MI 48109 USA; 4 Institute of Gerontology, University of Michigan, 1415 Washington Heights, M3007 SPH II, Ann Arbor, MI 48109 USA; 5 Department of Public Health & Biostatistics Consulting Center, Chang Gung University, 259 Wenhua 1st Road, Guishan District, Taoyuan, 33302 Taiwan; 6 Department of Orthopedic Surgery, Chang Gung Memorial Hospital, Linkou, 5 Fu-Hsing Street, Guishan District, Taoyuan, 33305 Taiwan; 7 Department of Internal Medicine, Chang Gung Memorial Hospital, Linkou, 5 Fu-Hsing Street, Guishan District, Taoyuan, 33305 Taiwan; 8 School of Nursing, College of Medicine, and Healthy Aging Research Center, Chang Gung University, 259 Wenhua 1st Road, Guishan District, Taoyuan, 33302 Taiwan; 9 Department of Nursing, Kaohsiung Chang Gung Memorial Hospital, 123 Dapi Road, Niaosng District, Kaohsiung, 83301 Taiwan; 10 Department of Gerontological Care and Management, Chang Gung University of Science and Technology, 261 Wenhua 1st Road, Guishan District, Taoyuan, 33303 Taiwan

**Keywords:** American Society of Anesthesiologists scores, Asa, Health-related quality of life, Hip-fractured adults, Mortality, Physical function recovery, Service utilization

## Abstract

**Background:**

Little is known about the relationship of the American Society of Anesthesiologists Physical Status Classification scores (ASA scores) on patient outcomes following hip fracture surgery in Asian countries. Therefore, this study explored the association of patients’ preoperative ASA scores on trajectories of recovery in physical functioning and health outcomes during the first year following postoperative discharge for older adults with hip-fracture surgery in Taiwan.

**Methods:**

The data for this study was generated from three prior studies. Participants (*N* = 226) were older hip-fracture patients from an observational study (*n* = 86) and two clinical trials (*n* = 61 and *n* = 79). Participants were recruited from the trauma wards of one medical center in northern Taiwan and data was collected prior to discharge and at 1, 3, 6, and 12 months after hospital discharge. Participants were grouped as ASA class 1–2 (50.5%; ASA Class 1, *n* = 7; ASA Class 2, *n* = 107) and ASA class 3 (49.5%, *n* = 112). Measures for mortality, service utilization, activities of daily living (ADL), measured by the Chinese Barthel Index, and health related quality of life, measured by Medical Outcomes Study Short Form-36, were assessed for the two groups. Generalized estimating equations (GEE) were used to analyze the changes over time for the two groups.

**Results:**

During the first year following hip-fracture surgery, ASA class 1–2 participants had significantly fewer rehospitalizations (6%, *p* = .02) and better scores for mental health (mean = 70.29, standard deviation = 19.03) at 6- and 12-months following discharge than those classified as ASA 3. In addition, recovery of walking ability (70%, *p* = .001) and general health (adjusted mean = 58.31, *p* = .003) was also significantly better than ASA 3 participants.

**Conclusions:**

There was a significant association of hip-fracture patients classified as ASA 1–2 with better recovery and service utilization during the first year following surgery. Interventions for hip fractured patients with high ASA scores should be developed to improve recovery and quality of life.

## Background

The worldwide incidence of hip fracture in 2000 was 1.6 million and is expected to rise dramatically as of 2050 to approximately 6.3 million, due to the ongoing aging of the world’s population [1]; an estimated 50% of those hip fractures are expected to occur in Asian populations [2]. Hip fracture affects the life expectancy and mortality of persons aged 65 years and older [3]. The mortality rate for elderly patients with hip fracture has been estimated to be between 21.5% to 27.3% within 1 year of fracture [3, 4]. In a previous study the 1-year mortality rate of elderly Taiwanese persons with hip fracture was found to be 15.5%, with only 56.1% recovering their previous performance of activities of daily living (ADL) and 37.9% recovering their instrumental ADL (IADL) 1 year following hip fracture [5].

One widely used measure of preoperative function is the American Society of Anesthesiologists Physical Status Classification (ASA class), which has been found to predict patient outcomes after hip fracture. For example, patients with an ASA class of 1 or 2 (healthy or with mild systemic illness, respectively) were more likely to be living at home 1 year after hip fracture [6, 7]. Similarly, 1-year mortality following surgery for hip fracture was almost 9-times higher for patients with an ASA class of 3 or 4 (severe systemic illness) than patients with an ASA class 1 or 2 [8]. In addition, higher ASA grades were associated with poorer 30-day outcomes, which included higher mortality, number of comorbidities, and number of inpatient readmissions [9, 10]. Higher ASA scores have also been associated with greater pain, less distance walked and poorer movement [11].

Currently, there is little knowledge regarding the correlation between preoperative ASA classes of hip fracture for persons in Asian countries and patient outcomes. Although the ASA score has been correlated with several factors regarding patient outcomes [2, 12] no studies have explored the association between ASA scores and the trajectory of recovery or longitudinal changes in health outcomes following hip fracture surgery in Taiwan [13]. Therefore, the aim of the present study was to determine the association, if any, of the preoperative ASA classes of a sample of older hip fracture patients in Taiwan on recovery trajectories as measured by physical functioning and health outcomes over the course of the first year following hospital discharge. We hypothesized that during the first year after surgical treatment for hip fracture, older patients with severe preoperative systemic disease (ASA score 3 and 4) would have higher mortality as well as increased service utilization, emergency room visits, and institutionalization. In addition, we hypothesized these patients would have poorer recovery in walking ability, performance of ADLs, and health-related quality of life than those with mild systemic disease (ASA grade 1 and 2) or healthy patients.

## Methods

### Data source and participants

This was a secondary analysis of data from three previously published studies of 285 participants recruited from the trauma wards of a medical center in northern Taiwan. We excluded 59 of the participants due to incomplete data relevant to this study. The hip-fracture participants for this analysis (*N* = 226) were from an observational study (*n* = 86 [13]), and control groups were non-interventional participants from two clinical trials (*n* = 61 [14] and *n* = 79 [15]). The Chang Gung Memorial Hospital Research Ethics Board approved these studies (NHRI-EX92-9023PL: CMRP819, 89–25, and 94-422c) and written informed consent was obtained for each participant. Data for these three studies were collected at baseline (prior to hospital discharge) and at 1-, 3-, 6-, and 12-month post-discharge. For all three studies, the inclusion criteria for hip-fracture patients were as follows: 1) age ≥ 60 years, 2) hospitalization resulting from a hip fracture that was subsequently treated with internal fixation or arthroplasty, 3) a place of residence located in northern Taiwan, and 4) a lack of any severe cognitive impairment as determined by a physician or by a score of ≥10 on the Chinese Mini-Mental State Examination (CMMSE) [16]. Because data was collected from self-report measures, patients found to have severe cognitive impairment were excluded if they were unable to complete the measures.

### Measures

#### ASA score

An anesthesiologist, aware of the study, assessed participants’ ASA scores, which was determined by the participant’s medical history. In Taiwan, ASA scores are divided into six classes: a completely healthy patient is given a class 1 ASA score; a patient with mild systemic disease is considered class 2; a patient with a severe but not incapacitating systemic disease is class 3 ASA; a patient with an incapacitating disease that is also life-threatening is class 4 ASA; and a moribund patient not expected to live for 24 h with or without surgery is class 5. A class 5 ASA score is also taken to indicate a need for emergency surgery. A class 6 ASA score is provided to patients classified as brain-dead and eligible to be designated as organ donors. The validity of using ASA scores to indicate a patient’s long-term mortality and complications following total hip arthroplasty [6, 17] and hip-fracture surgery [8] has been well established. ASA scores are routinely used in Taiwan to assess for anesthesia and surgery risk.

#### Activities of daily living (ADL)

The Chinese Barthel Index (CBI) was used to measure each patient’s ability to perform ADL. The CBI exhibits satisfactory reliability and validity when used as an assessment tool for assessing Taiwanese older adults with hip fracture [18]. Specifically, the CBI is used to measure the level of dependency of a patient with regard to each of the following activities: bathing, grooming, dressing, eating, using the toilet, transferring, climbing stairs, and bowel/bladder control. CBI scores range from 0 (indicating complete dependence on assistance from others in performing all of the ADL) to 100 (indicating complete independence from others in performing all of the ADL).

#### Recovery of walking ability

The recovery of each patient’s walking ability was evaluated using the item of the CBI that assesses independence in walking; a comparison was made between the patient’s postoperative walking ability score and his or her score prior to the fracture, with said pre-facture walking ability being assessed retrospectively according to the patient’s recall at the time of admission. If the patient’s postoperative walking ability score was the same as or greater than his or her walking ability before the fracture, it was coded as 1, whereas it was coded as 0 if it was more limited than his or pre-fracture walking ability [5, 19].

#### Health-related quality of life (HRQoL)

The Taiwan version of the Medical Outcomes Study Short-Form 36 (SF-36) [15, 19, 20] was used in order to assess each patient’s HRQoL. The SF-36 includes a total of 36 items used to assess a variety of health-related domains: bodily pain (BP), physical functioning (PF), vitality (VT), the role of disability due to physical health problems (RP), and general perceptions of health. Among elderly hip-fracture patients who are independent before their hip fracture, approximately half become partially or wholly dependent on others after the fracture in terms of social functioning (SF), self-care ability [5], general mental health, and role disability due to emotional problems (RE). The total score for each of these relevant sub-scales range from 0 to 100, with better health outcomes being indicated by higher scores.

### Procedures

The Committee on Human Research at the hospital approved all three studies, all of which were conducted using the same research procedure. Specifically, research assistants identified possible study participants subsequent to each patient’s surgery but before each patient was discharged from the hospital. After a potential participant was identified, a research assistant then invited him or her to participate in the given study. Patients who agreed to take part in the study then signed a written informed consent, after which they were assessed at five different time points: prior to hospital discharge, and at 1, 3, 6, and 12 months after discharge. Relevant covariate data, such as demographic data and data on cognitive functioning, were collected from the patients prior to hospital discharge.

#### Covariates

In the analysis for predictors of outcome variables, we controlled for covariates of time, gender, age, type of surgery (internal fixation or arthroplasty), pre-fracture performance of ADLs, and data set membership (study of origin). Pre-fracture ADL performance was measured by patients’ self-reported CBI score. To control for possible significant differences among the three data sets, they were identified as covariates by including two dummy variables in the regression analysis of generalized estimating equations (GEE).

##### Statistical analysis

Data analysis was performed using Statistical Analysis Software [21]. The overall participant sample consisted of patients with ASA scores from 1 to 3. Participants were sub-divided into two groups: healthy or mild systemic disease (class 1–2) and severe systemic disease (class 3) [8]. For demographic variables such as gender, educational background, mortality, institutionalization and emergency room visits, Chi-square tests compared the differences between the two groups. Student’s t-test, two-tailed, compared the differences in continuous variables between the two groups. GEE analysis determined whether patients could be expected to recover pre-fracture abilities for ADL, walking, and HRQoL in the first 12 months after hospital discharge. GEE can account for possible correlations in repeated measures over time and can explore the differences at different time points. In longitudinal studies, GEE analysis is especially useful for data belonging to participants who die or are lost to follow up; analysis can be applied to attrition without using imputation [22]. Therefore, data for participants with at least one observation were included in the analysis, if participants died or were lost to follow up during the year post-discharge. The participants with at least one observation were analyzed by the GEE approach, which included a total of 114 participants in the ASA 1–2 group, and 112 participants in the ASA 3 group. The number of participants remaining at each time point for each group are listed in Table 2. Time, gender, age, type of surgery, pre-fracture ADL performance data set membership was controlled for as a covariate in the GEE analysis [21]. Level of significance was set at *p* < .05 [23].

## Results

### Participant characteristics

Participants consisted of patients with ASA scores from 1 to 3. Comparison of hip-fractured older adults classified as ASA 1–2 and ASA 3 showed no significant differences in demographics, with the exception of age (Table 1). Significant differences for only two clinical characteristics were seen for participants classified as ASA 3: number of comorbidities (*p* = .024) and the presence of heart disease (*p* = .04) (Table 1).

**Table 1:** Demographic and clinical characteristics for all hip-fractured participants, and differences between groups: preoperative ASA scores determined by independent t-test (a) or Chi-squared tests

	Total Participants	Groups		
Variable	(*n* = 226)	ASA 1–2 (*n* = 114)	ASA 3 (*n* = 112)	*p*-Value
Age (years) (mean ± SD)	78.22 ± 7.70	77.06 ± 7.40	79.39 ± 7.85	.023^a^
Gender, *n* (%)				.14
Male	78 (34.5%)	34 (29.8%)	44 (39.2%)	
Female	148 (65.5%)	80 (70.2%)	68 (60.7%)	
Hospital stay (days) (mean ± SD)	9.96 ± 5.06	10.02 ± 4.91	9.89 ± 5.24	.85
Education, *n* (%)				.92
None	133 (58.5%)	69 (60.6%)	64 (57.1%)	
Primary	52 (23.0%)	24 (21.1%)	28 (25.0%)	
High School	27 (11.9%)	14 (12.3%)	13 (11.6%)	
College or above	14 (6.2%)	7 (6.1%)	7 (6.2%)	
Fracture classification, *n* (%)				.73
Femoral neck	113 (50.0%)	57 (50.0%)	56 (50.0%)	
Intertrochanteric	104 (46.0%)	52 (45.6%)	52 (46.4%)	
Subtrochanteric	9 (4.0%)	5 (4.4%)	4 (3.6%)	
Type of surgery, *n* (%)				.76
Arthroplasty	87 (38.5%)	45 (39.5%)	42 (37.5%)	
Internal fixation	139 (61.5%)	69 (60.5%)	70 (62.5%)	
Number of comorbidities (mean ± SD)	0.90 ± 0.85	0.77 ± 0.86	1.03 ± 0.82	.024^a^
Presence of comorbidity (type), *n* (%)				
Cardiovascular disease	46 (20.4%)	17 (14.9%)	29 (25.9%)	.04
Hypertension	99 (43.8%)	46 (40.4%)	53 (47.3%)	.29
Diabetes mellitus	58 (25.7%)	25 (21.0%)	33 (29.7%)	.20
Unable to walk independently pre-fracture, n (%)	17 (7.5%)	5 (4.4%)	12 (10.7%)	.07
CBI performance, pre-fracture (mean ± SD)	97.15 ± 6.39	97.72 ± 5.84	96.56 ± 6.88	.174^a^

*Abbreviations*: *ASA* American Society of Anesthesiologists; *SD* standard deviation; *CBI* Chinese Barthel Index

^a^ *P*-value determined by independent t-test, otherwise determined by Chi-square test

### Differences in service utilization and mortality

Comparisons of outcomes for participants in the ASA 1–2 and ASA 3 groups at 1-, 3-, 6-, and 12-months following hospital discharge are shown in Table 2, including numbers of remaining participants at each time point for each group. The number of participants readmitted to the hospital at 12 months was significantly greater for those classified as ASA 3 than those classified as ASA 1–2 (*p* = .02). However, there was no significant difference in emergency room visits or mortality rates for these two groups in the first year after discharge (Table 2).

**Table 2:** Comparison of outcomes for participants grouped by preoperative ASA scores following hip-fracture surgery within 1-,3-, 6- and 12-month post-discharge

	Participant Group		
Outcome Post-discharge	ASA 1–2	ASA 3	*p*-Value^a^
Participants within 1 month, n (% remaining)	114 (100%)	112 (100%)	
Mortality	0 (0.0%)	0 (0.0%)	
Institutionalization	2 (1.8%)	7 (6.3%)	.08
Emergency room visits	7 (6.1%)	9 (8.1%)	.57
Hospital readmissions	6 (5.3%)	13 (11.6%)	.09
Recovery of walking ability	55 (48.2%)	33 (29.5%)	.004
CBI^b^ score (mean ± SD)	73.46 ± 19.68	68.57 ± 19.43	.06
HRQOL^c^ Subscale Scores (mean ± SD)			
Bodily pain	69.58 ± 24.47	71.96 ± 25.09	.47
General health	62.16 ± 23.41	50.02 ± 25.46	.006
Vitality	57.86 ± 24.55	58.38 ± 24.27	.87
Social functioning	62.61 ± 30.69	61.48 ± 29.82	.78
Role disability/emotional	66.07 ± 44.29	66.97 ± 44.09	.88
Mental health	62.40 ± 22.58	63.09 ± 19.78	.81
Physical functioning	33.93 ± 38.13	28.64 ± 38.43	.30
Role disability/physical	28.57 ± 42.82	22.48 ± 37.88	.26
Participants within 3 months, n (% remaining)	108 (94.7%)	107 (95.5%)	
Mortality	1 (0.9%)	2 (1.8%)	.55
Institutionalization	3 (2.8%)	4 (3.7%)	.71
Emergency room visits	5 (4.6%)	4 (3.7%)	.73
Hospital readmissions	4 (3.7%)	5 (4.6%)	.73
Recovery of walking ability	79 (73.8%)	53 (48.6%)	< .001
CBI^b^ score (mean ± SD)	83.19 ± 17.94	80.09 ± 17.52	.20
HRQOL^c^ Subscale Scores (mean ± SD)			
Bodily pain	72.10 ± 23.12	75.52 ± 24.89	.31
General health	58.62 ± 24.26	52.56 ± 24.14	.07
Vitality	61.94 ± 21.33	62.43 ± 22.17	.87
Social functioning	59.98 ± 24.60	62.14 ± 28.25	.56
Role disability/emotional	79.17 ± 38.36	81.88 ± 35.78	.60
Mental health	67.25 ± 19.49	65.18 ± 21.44	.47
Physical functioning	31.35 ± 25.35	26.36 ± 26.15	.17
Role disability/physical	20.91 ± 34.33	25.73 ± 38.58	.34
Participants within 6 months, n (% remaining)	102 (89.5%)	97 (86.6%)	
Mortality	2 (1.9%)	4 (3.7%)	.41
Institutionalization	3 (3.0%)	3 (3.1%)	.97
Emergency room visits	9 (8.9%)	10 (10.5%)	.70
Hospital readmissions	7 (6.9%)	11 (11.6%)	.26
Recovery of walking ability	75 (73.5%)	64 (64.6%)	0.17
CBI^b^ score (mean ± SD)	85.49 ± 22.37	86.13 ± 17.39	.82
HRQOL^c^ Subscale Scores (mean ± SD)			
Bodily pain	78.10 ± 24.69	83.74 ± 22.28	.10
General health	60.11 ± 23.09	57.72 ± 23.87	.49
Vitality	63.68 ± 21.38	65.16 ± 21.90	.64
Social functioning	73.08 ± 26.54	75.41 ± 27.60	.56
Role disability/emotional	85.51 ± 32.89	88.30 ± 30.80	.55
Mental health	68.04 ± 20.57	68.09 ± 18.98	.95
Physical functioning	55.75 ± 32.47	57.23 ± 34.75	.76
Role disability/physical	33.60 ± 40.96	47.85 ± 45.54	.03
Participants within 12 months, n (% remaining)	84 (73.7%)	81 (72.3%)	
Mortality	3 (3.1%)	8 (8.1%)	.13
Institutionalization	1 (1.2%)	3 (3.6%)	.31
Emergency room visits	3 (3.6%)	8 (10.0%)	.10
Hospital readmissions	5 (6.0%)	14 (17.3%)	.02
Recovery of walking ability	64 (76.2%)	56 (67.5%)	.21
CBI^b^ score (mean ± SD)	85.71 ± 26.94	87.04 ± 19.55	.71
HRQOL^c^ Subscale Scores (mean ± SD)			
Bodily pain	70.04 ± 26.46	77.75 ± 25.83	.08
General health	53.77 ± 23.67	50.60 ± 25.24	.45
Vitality	60.59 ± 20.25	59.93 ± 21.91	.85
Social functioning	74.30 ± 27.13	66.37 ± 30.30	.10
Role disability/emotional	91.30 ± 25.98	82.87 ± 37.11	.12
Mental health	70.29 ± 19.03	63.14 ± 22.70	.047
Physical functioning	50.70 ± 29.76	41.99 ± 31.97	.09
Role disability/physical	45.07 ± 43.02	38.19 ± 45.57	.36

^a^ *p*-value determined by student’s t-test, two-tailed

^b^CBI (Chinese Barthel Index): higher score indicates greater independence

^c^HRQOL (Health Related Quality of Life); higher score indicates better health outcomes

### Relationship between ASA score, recovery of physical function, and HRQoL

Outcomes for recovery of walking ability, ADLs, as measured by CBI scores, and scores for subscales of HRQoL were compared for the two groups at the four times post-discharge (Table 2). Participants with ASA 3 had poorer recovery of walking ability at 1- and 3-months compared with participants classified as ASA 1–2 (*p* = .004 and *p* < .001, respectively). There was no significant difference in CBI scores between groups at any time post-discharge. Differences in some of the subscale scores for HRQoL were significantly lower at various times post-discharge for the ASA 3 group: general health at 1 month (*p* = .006); mental health at 12-months; and role disability/physical at 6-months.

**Fig. 1:**
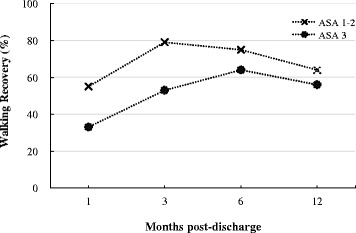
Trajectory of hip-fracture surgery patients’ walking recovery relative to preoperative ASA scores of 1–2 and ASA 3 over time after hospital discharge. (ASA: American Society for Anesthesiologists)

**Fig. 2:**
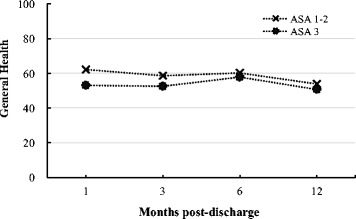
Trajectory of hip-fracture surgery patients’ recovery of general health over time after hospital discharge relative to groups with preoperative ASA scores of 1–2 and ASA 3. (ASA: American Society of Anesthesiologists)

These differences between groups were examined by GEE analysis, controlling for covariates of time, gender, age, type of surgery, and pre-fracture CBI performance data set membership (Table 3). The participants in the group with preoperative ASA 1–2 scores had significantly better scores for walking ability recovery (beta coefficient = 0.38, *p* = .001, Table 3, and Fig. 1) than the ASA 3 group, indicating better walking ability recovery during the first year following discharge. Moreover, the ASA 1–2 group had significantly better outcomes for general health (beta coefficient = −4.95, *p* = .032, Table 3, and Fig. 2) than the ASA 3 group, indicating better overall outcomes in general health during the first year following discharge. However, the ASA 3 group had significantly better scores for coping with bodily pain (beta coefficient = 4.86, *p* = .028,) than the ASA 1–2 group.

**Table 3:** Association between participants’ preoperative ASA score classification and recovery of physical and mental functioning during the first year following hospital discharge for hip-fracture surgery: General Estimating Equation (GEE) analysis

	Time (months post-discharge)							Group		
Outcome variable	1	3^a^		6^a^		12^a^		ASA 1-2^b^		ASA 3
	(%)/Mean	β	(%)/Mean	β	(%)/Mean	β	(%)/Mean	(%)/Mean	β	(%)/Mean
Walking recovery, %^c^	39%	0.97	63%^§^	1.30	70%^§†^	1.4	73%^§†^	70%	0.38	53%^#^
CBI score, mean^d^	71.91	10.27	82.18^§^	13.86	85.77^§†^	1.39	85.85^§†^	81.24	13	81.62
HRQOL scores, mean^d^										
BP	71.35	2.95	74.31	10.08	81.43^§†^	2.83	74.18^•^	72.89	4.86	77.75^#^
GH	58.06	−2.47	55.60	0.64	58.71	−7.09	50.98^§†•^	58.31	−4.95	53.36^#^
VT	57.8	3.56	61.37	6.05	63.85^§^	1.43	59.24^•^	59.85	1.43	61.28
SF	62.76	−0.93	61.50	12.06	74.49^§†^	7.60	70.63^§†^	67.25	−0.28	66.97
RE	66.88	13.99	80.86^§^	19.94	86.81^§†^	18.08	85.68^§^	79.65	0.82	80.47
MH	62.76	2.95	65.72	4.91	67.67^§^	2.79	65.55	65.89	−0.93	64.96
PF	32.72	−2.45	30.27	24.22	56.94^§†^	11.64	44.36^§•^	43.05	−3.95	39.10
RP	27.39	−2.46	24.93	13.83	41.23^§†^	12.14	39.54^§†^	32.81	0.93	33.74

*Abbreviations*: *CBI* Chinese Barthel Index, (activities of daily living); HRQOL = health related quality of life; *BP* bodily pain; *GH* general health perception; *VT* vitality; *SF* social functioning; *RE* role disability due to emotional problems; *MH* general mental health; *PF* physical functioning; *RP* role disability due to physical health problems

^a^Time post-discharge compared with 1 month

^b^Participant group ASA 1–2 compared with ASA 3

^c^Percentage was calculated only for categorical outcome variables

^d^Mean = adjusted mean obtained after controlling for time, gender, age, type of surgery, and pre-fracture CBI performance data set membership

^#^Indicates a significant difference between ASA 1–2 and ASA 3 group, *p* < .05

^§^Indicates time post-discharge was significantly different compared with 1 month for both ASA 1–2 and ASA 3, *p* < .05

^†^Indicates a significant difference compared with 3 months for both ASA 1–2 and ASA 3, *p* < .05

^•^Indicates a significant difference compared with 6 months for both ASA 1–2 and ASA 3, *p* < .05

## Discussion

To our knowledge, this is the first study to evaluate the relationship between preoperative ASA scores and patient outcomes during one-year following hip-fracture surgery for older adults in Taiwan. ASA classifications were associated with levels of service utilization, mortality, and longitudinal changes in both physical recovery and various aspects of HRQoL.

Our results demonstrated higher preoperative ASA scores correlated with higher hospital readmissions at 6- and 12-months following hospital discharge, which is consistent with previous reports showing higher preoperative ASA scores are associated with more hospital readmissions [9, 10] and poorer ambulatory recovery [10, 24]. Our findings showed participants with ASA scores of 3 had significantly poorer recovery of walking ability at 1-and 3-months after discharge. However, although post-discharge ADL performance (CBI score) differed at 1-month post-discharge between those classified as ASA 1–2 and ASA 3, there was no difference in long-term outcomes at 1-year, which is consistent with previous findings [8, 25]. However, for the total sample of participants, with no grouping by ASA scores, walking recovery and CBI scores gradually improved over time in the 1 to 12 months after surgery, demonstrating recovery was proportional to time.

Quality of life also gradually improved over time during the first 1- to 6-months following surgery. However, quality of life decreased at 12 months. One explanation may be that physical recovery improved more rapidly during the first 6 months, which has been shown to influence a perception of an improvement in quality of life [5]. However, as the speed of recovery slowed in the latter half of the year following surgery [5], most dimensions for health-related quality of life had reached a plateau [26]. Hip-fractured patients with ASA scores of 3 also had poorer health outcomes in several subscales for HRQoL at different times post-discharge, as well as the overall trajectories of these subscales, which has not previously been reported. This finding adds new knowledge regarding the relationship of ASA classifications to HRQoL scores.

We found no significant difference in mortality between participants with high or low ASA scores, which is in contrast to previous findings [3, 8]. This might be due to the health and independence of our population. For example, approximately 61.3% of participants in a previous study experienced severe systemic disease (ASA 3 & 4) [10], whereas none of our study participants were classified as having severe (class 4) systemic disease. Therefore, although the mortality rate in our study was higher for the ASA 3 group (12.5%) than the ASA 1–2 group (5.3%), we might need a larger sample to achieve a statistically significant difference.

In addition, older patients with high and low preoperative ASA scores [8] have been shown to differ significantly before hip-fracture surgery in the percentages with cardiovascular diseases, diabetes mellitus and walking difficulty. In our study, although greater percentages of participants with higher ASA scores had hypertension, cardiovascular diseases and difficulty in walking before hip-fracture, only cardiovascular disease was statistically significant, which is similar to a previous report showing a correlation between ASA scores and cardiovascular disease [8–10].

The participants in our study were relatively independent before their hip-fracture, with only 6.2% having difficulty walking, whereas 29.2% of participants in a previous study had difficulty walking [8]. However, participants with ASA 3 scores appeared to have significantly better control of coping with bodily pain than the ASA 1–2 group. This finding should be further explored with a larger sample, due to the small variances in independence and health conditions in our sample. Our findings highlight the need to focus on older patients with high preoperative ASA scores during the first year after hospital discharge following hip-fracture surgery, particularly with regards to strategies for preventing hospital readmissions. This should include intervention protocols, which are developed specifically for patients with higher preoperative ASA scores.

## Study limitations

The generalizability of the study findings is limited by the use of secondary data as well as the study’s utilization of a relatively small convenience sample, the fact that some of the study participants were lost to follow-up, and the lack of participation by patients with severe physical limitations (ASA Class 4). In Taiwan, patients that are Class 4 or higher have an incapacitating disease that is also life-threatening, and thus they seldom receive surgery. Thus, the study findings can only be generalized to that portion of the population of older Taiwanese hip fracture patients who exhibited independence prior to their fractures. Finally, the original studies did not collect information on the specifics of the hip-fracture surgery. Therefore, our secondary analysis did not include data on type of surgical reduction performed for hip-fracture: closed reduction with a sliding hip screw; closed reduction with IM nail; Internal fixation without reduction and with cancellous screws; and open reduction and dynamic condylar screws. The type of surgery may affect outcomes [27, 28] and this was not accounted for in our study. Therefore, we suggest future studies include detailed information regarding the type of surgery performed.

## Conclusion

We found higher ASA scores were associated with more hospital readmissions at 1- and 12-months following hospital discharge. In addition, better recovery of walking ability and general health was associated with participants having ASA scores of I-II during the first year following hip-fracture surgery. The present study and its findings could serve as a valuable reference for health care professionals in countries providing care to older adult Chinese/Taiwanese hip-fracture patients.

## Abbreviations

ADL: Activities of daily living
ASA class: American Society of Anesthesiologists Physical Status Classification
ASA: American Society of Anesthesiologists
BP: bodily pain
CBI: Chinese Barthel Index
CMMSE: Chinese Mini-Mental State Examination
GEE: generalized estimating equations
HRQoL: Health-related quality of life
IADL: instrumental ADL
MOS SF-36: Medical Outcomes Study Short Form 36
PF: physical functioning
RE: role disability due to emotional problems
RP: role of disability due to physical health problems
SF: social functioning
VT: vitality
